# The multi-domain long term care needs and unmet needs of Veterans with a stroke diagnosis

**DOI:** 10.1093/geroni/igaf122.4029

**Published:** 2025-12-31

**Authors:** Roshni Singh, Diana Ruiz, Richard Munoz, Marianne Desir, Sandra Garcia-Davis

**Affiliations:** University of Miami, Miami, Florida, United States; Miami Veterans Affairs Hospital, Miami, Florida, United States; South Florida Veteran Affairs Foundation for Research and Education, Miami, Florida, United States; Miami Veterans Affairs Hospital, Miami, Florida, United States; Nova Southeastern University, Fort Lauderdale, Florida, United States

## Abstract

Stroke survivors frequently experience several unmet needs, which can significantly impact quality of life and ability to age in the community. We used data from the HERO CARE survey, fielded in 2021 to 20,000 community-dwelling Veterans across five VA sites (respondent N = 8,056). Veterans with a history of stroke diagnosis were defined by Hierarchical Condition Category 100 (n = 576). We sought to describe the long-term care needs of stroke survivors across five domains: activities of daily living (ADLs), instrumental ADLs (IADLs), nursing, health care coordination, and social (legal and housing). We classified respondents as having an unmet need if they responded that they needed a little or a lot more help for a specific need. Veteran respondents were mostly men (97.7%), average age 80.4 (SD 8.7), married (61.3%), Non-Hispanic White (74.1%), residing in urban areas (81.9%), low health literacy (61.8%), and with a caregiver (70.4%). Veterans reported difficulties with ADLs (69.3%), IADLs (79.0%), nursing tasks (70.6%), health care coordination (72.2%), and social (48.5%). Overall, 54.2% reported having an unmet need, with ADLs (29.5%), IADLs (38.6%), nursing (29.4%), health care coordination (30.3%), and social (20.5%). Veterans also reported psychological and social concerns: dementia (29%), possible depression (29%), possible anxiety (18.8%), food insecurity (19.1%), and transportation problems (17.1%). Stroke survivors describe complex unmet needs. They require proactive, comprehensive support to address their multi-domain long-term care needs to empower them to live in the community. These data can aid in developing programs and policies to support stroke survivors and their caregivers.

